# Myokine/Adipokine Response to “Aerobic” Exercise: Is It Just a Matter of Exercise Load?

**DOI:** 10.3389/fphys.2019.00691

**Published:** 2019-05-29

**Authors:** Zihong He, Ye Tian, Pedro L. Valenzuela, Chuanye Huang, Jiexiu Zhao, Ping Hong, Zilin He, Shuhui Yin, Alejandro Lucia

**Affiliations:** ^1^Biology Center, China Institute of Sport Science, Beijing, China; ^2^Culture Development Center, General Administration of Sport of China, Beijing, China; ^3^Physiology Unit, Department of Systems Biology, University of Alcalá, Madrid, Spain; ^4^Graduate School, Shandong Sport University, Jinan, China; ^5^Winter Sports Administrative Center, General Administration of Sport of China, Beijing, China; ^6^Cardiovascular Department, Beijing Jian Gong Hospital, Beijing, China; ^7^The Rocket Force General Hospital of PLA, Institute of Hepatobiliary Gastrointestinal Disease, Beijing, China; ^8^Faculty of Sport Sciences, European University of Madrid, Madrid, Spain; ^9^Institute Hospital 12 de Octubre, Madrid, Spain

**Keywords:** cytokines, metabolism, physical activity, training, responders

## Abstract

**Purpose:**

Exercise health benefits are partly mediated by exertional changes in several myokines/adipokines. This study aimed to compare the acute response of some of these biomarkers to aerobic exercise performed at the intensity corresponding to the maximum fat oxidation rate (FATmax) or the “anaerobic” threshold (AT).

**Methods:**

Following a cross-over, counterbalanced design, 14 healthy untrained men (23 ± 1 years) performed a 45-min exercise bout at their FATmax or AT intensity (been previously determined through incremental exercise tests). The concentration of interleukin (IL)-15, follistatin, myostatin, fibroblast-growth factor (FGF)-21, irisin, resistin, and omentin was measured at baseline and 0, 1, 3, 24, 48, and 72 h post-exercise.

**Results:**

AT exercise was performed at a higher intensity (85 ± 8 vs. 52 ± 14% of maximal oxygen uptake [VO_2 max_], *p* < 0.001) and induced a higher energy expenditure (*p* < 0.001) than FATmax, whereas a greater fat oxidation was observed in the latter (*p* < 0.001). A higher peak response of FGF-21 (+90%, *p* < 0.01) and follistatin (+49%, *p* < 0.05) was found after AT-exercise, as well as a trend toward a higher peak level of omentin (+13%, *p* = 0.071) and a greater decrease in resistin (−16%, *p* = 0.073).

**Conclusion:**

Increasing exercise load (from FATmax to AT) results in a higher response of FGF-21, follistatin and omentin to aerobic exercise, with the subsequent potential cardiometabolic benefits. No effects were, however, observed on the remainder of biomarkers. Future research should address if manipulating other exercise variables (e.g., type, frequency) can promote a higher myokine/adipokine response.

## Introduction

Regular physical exercise is an effective lifestyle intervention for the prevention and treatment of numerous non-communicable diseases (Fiuza-Luces et al., 2013, 2018), with “aerobic” (or “endurance”) exercise (e.g., brisk walking, jogging/running, swimming) being probably the most commonly prescribed modality (Warburton et al., 2006). Aerobic exercise has proven to reduce cardiovascular disease (CVD) risk factors such as high blood pressure, hyperlipidemia, or altered glucose homeostasis, among others (Whelton et al., 2002; Snowling and Hopkins, 2006; Kodama et al., 2007). However, the magnitude of the health benefits seem to depend on exercise loads (Duncan et al., 2005). For instance, a greater improvement in CVD risk factors and cardiorespiratory fitness (CRF) might be observed with vigorous exercise (i.e., >6 metabolic equivalents [METs], or >60% of maximal oxygen uptake [VO_2 max_]) than with less intense training (Swain and Franklin, 2006).

The prescription of aerobic training loads can therefore be modified to maximize health benefits. In this regard, there is a high inter-individual variability in the physiological responses and adaptations to exercise at a fixed relative intensity (i.e., expressed as a percentage of VO_2 max_ or maximal heart rate) (Mann et al., 2013). By contrast, prescribing exercise loads relative to individually determined specific physiological indicators (“thresholds”) whose relative intensity varies between individuals might homogenize the elicited stress and thus reduce individual variability in metabolic responses (Mann et al., 2013). For instance, a commonly prescribed intensity is that associated to the so-called “anaerobic threshold” [AT, although the term “anaerobic” is physiologically inappropriate (Chamari and Padulo, 2015)], which is also termed “second ventilatory threshold” or “respiratory compensation point” (Meyer et al., 2005), and represents the maximum intensity that can be maintained in a steady state oxidative metabolism (Connolly, 2012). On the other hand, exercise prescription at the intensity corresponding to the point of maximal fat oxidation (FATmax) has gained popularity during the last decade (Jeukendrup and Achten, 2001). The latter strategy has proven beneficial for weight management and for improving several markers of cardiometabolic health (Venables and Jeukendrup, 2008; Romain et al., 2012).

Although both AT and FATmax training provide cardiometabolic benefits, the results of comparing these two exercise intensities remain to be reported. In this respect, the benefits of aerobic training on cardiometabolic health seem to be partly mediated by the cumulative effects of repeated, acute bouts of exercise-induced changes in several hormones and molecules such as the so-called myokines and adipokines, which are released from muscles and adipose tissue, respectively, to the blood and exert endocrine or paracrine effects in other cells, tissues or organs (Pedersen and Febbraio, 2012; Fiuza-Luces et al., 2018).

Several myokines have been proposed to mediate exercise-induced health benefits besides the most studied and well known myokine, interleukin (IL)-6 (Fiuza-Luces et al., 2013). Notably, IL-15, fibroblast growth factor (FGF)21, or irisin have been reported to induce cardiometabolic benefits through promoting the browning of white adipose tissue (WAT), and enhancing glucose homeostasis (Busquets et al., 2006; Nielsen et al., 2008; Elbelt et al., 2013; Kim H. J. et al., 2013; Woo et al., 2013; Fisher and Maratos-Flier, 2016; Perakakis et al., 2017). Other myokines, mainly myostatin or follistatin, are more related to muscle plasticity, with the former being a negative regulator of muscle growth (Huang et al., 2011) and the latter promoting skeletal muscle development through the activation of anabolic pathways (Winbanks et al., 2012). Evidence suggests that these myokines also exert metabolic benefits (Huang et al., 2011; Braga et al., 2014). In addition, exertion-related changes in the circulating levels of adipokines such as omentin and resistin have been proposed to mediate the cardiometabolic benefits of exercise, with the former enhancing glucose metabolism (Yang, 2006), and the latter – which has been reported to decrease in response to exercise (Prestes et al., 2009) – antagonizing insulin action and being associated with obesity (Steppan et al., 2001; Adeghate, 2004).

Thus, the magnitude of release of these factors in response to exercise might partly explain the different benefits obtained depending on the training stimulus. However, evidence is still lacking regarding the influence of different training variables, notably relative intensity, on this response. The main purpose of this study was to compare the response of several myokines/adipokines involved in cardiometabolic health to an exercise session of the same total duration performed at either AT or FATmax intensity. The effect of these two types of training sessions on resting metabolic rate (RMR) was also analyzed as a secondary endpoint. We hypothesized that, since the AT usually corresponds to a higher intensity than FATmax (∼80–85 vs. 50–60% of VO_2 max_, respectively), an exercise session at AT-intensity would result in a higher total exercise load and promote a more marked release of myokines/adipokines than a FATmax-session, with the subsequent potential cardiometabolic health benefits.

## Materials and Methods

### Participants

Fourteen male subjects participated in this study ([mean ± SD] age, 23 ± 1 years; body mass index, 22 ± 2 kg ⋅ m^−2^). Inclusion criteria were being healthy (i.e., free of CVD, diabetes or abnormal glucose tolerance, or any other acute/chronic disease), aged 18 years or older, and performing no regular physical exercise (i.e., <20 min twice a week). Participants were required to maintain the same dietary habits during the study, and to refrain from doing exercise, smoking or drinking coffee or alcohol during the 48 h prior to each visit. They were informed of the objects and procedures and provided both verbal and written informed consent. The study was conducted in accordance with the Declaration of Helsinki and was approved by the Institutional Review Board (Ethics Committee of the Chinese Institute of Sport Science).

### Experimental Design and Measurements

Each participant attended our laboratory at 7.00 am on four different days after an overnight fast. Thereafter they ate a standardized breakfast (one cup of soy milk, one egg, and two ∼50 g steamed stuffed buns), and 2 h later they completed the exercise tests or sessions that are described below. All exercise tests and sessions were conducted on the same treadmill (pulsar4.0; H/P/cosmos; Traunstein, Germany).

#### Incremental Test for AT and VO_2 max_ Determination

The day of the first visit to the laboratory, participants performed an incremental treadmill exercise test in which speed was initially set at 7 km ⋅ h^−1^ and was increased by 1 km ⋅ h^−1^ every minute until volitional exhaustion, while treadmill inclination was kept constant at 0%. The test was deemed valid if at least three of the following four criteria were met: (a) a plateau in oxygen uptake (VO_2_) was observed despite increasing treadmill speed; (b) the subject was no longer able to maintain the required speed; (c) the respiratory exchange ratio (RER) exceeded a value of 1.10; and (d) the age-predicted maximum heart rate (HR_max_, 220 minus age, in years) was achieved. Gas-exchange data were consistently collected breath-by-breath with the same metabolic cart during the study (MetaMax 3B, Cortex, Biophysik; Leipzig, Germany). The VO_2 max_ was determined as the highest 30-s average VO_2_ value whereas the AT was calculated with three validated methods using 30-s average data (Meyer et al., 2005): (a) a modified V-slope for the relationship between ventilation (VE, in the *y* axis) and carbon dioxide production (VCO_2_, in the *x* axis), that is, a disproportionate increase in VE with respect to VCO_2_ (i.e., two regression lines are fitted for the upper and lower part of the relationship and their intersection represents the AT); (b) the ventilatory equivalent method (i.e., the first systematic rise in the ventilatory equivalent for CO_2_); and (c) the first decrease in the expiratory fraction of CO_2._ Two experienced researchers independently determined the AT by visual inspection using the three methods, and the mean of the three measurements was entered for analysis. Of note, the second two methods (b and c) do not usually provide additional information with respect to the first one other than a different representation of the onset of exercise induced-hyperventilation (Meyer et al., 2005).

#### Incremental Test for FATmax Determination

After 4–7 days, participants performed another incremental exercise test (protocol modified from Suk et al., 2015) for the determination of the intensity associated with their individual FATmax. After a 3-min warm-up at 20% of the velocity associated to the VO_2 max_ (V_max_), participants performed six 6-min stages at 25, 35, 45, 55, 65, and 75% of the V_max_, with a 5-min rest between stages. Mean fat expenditure (g/h) was calculated from the last 3 min of each stage, and FATmax was determined as the highest point of the binomial parabola formed by the fat expenditure-velocity data.

#### AT and FATmax Exercise Sessions and Subsequent Measurements

After another 4–7-day period, participants performed two 45-min “aerobic” exercise training sessions at either FATmax or AT intensity, following a cross-over, counterbalanced design, and with a 7-day rest period between sessions. Each session was preceded by a 10-min general warm up consisting of mobility exercises, walking, and jogging at a self-selected intensity. They were required to not surpass an intensity above which they could not talk easily. Aerobic energy expenditure during the training sessions was determined through the analysis of VO_2_ (MetaMax 3B, Cortex, Biophysik; Leipzig, Germany), and heart rate (HR) was also continuously assessed with a heart rate monitor (RS400, Polar, Kempele, Finland).

RMR was analyzed at baseline (before the standardized breakfast) and 24, 48, and 72 h after each session. During RMR measurements participants lied supine for 20 min in a room that had minimal light and noise, and constant ambient temperature (22 ± 1°C). Measurements were deemed valid if there was a variation <25 ml min^−1^. The first 3 min were considered as the stabilization phase and were discarded from the analyses.

Blood variables were analyzed at baseline, immediately upon session termination (post 0 h), and 1, 3, 24, 48, and 72 h after each session, respectively. Blood samples (10-mL) were drawn from the antecubital vein and centrifuged at 3000 × *g* for 10 min. The obtained serum was then kept at −80°C until analysis with enzyme-linked immunosorbent assays (ELISA) of: follistatin (number: DFN00, R&D Systems; Minneapolis, MN, United States); myostatin (number: DGDF80, R&D Systems); FGF-21 (number: DF2100, R&D Systems); IL-15 (number: 0707170149, R&D Systems); irisin (number: EK-067-29, Phoenix Pharmaceuticals, Burlingame, CA, United States); omentin (number: EZH0MNTN1-29K, Millipore, Burlington, MA, United States); and resistin (number: DRSN00, R&D Systems). The intra-assay coefficient of variation was ≤5.3, ≤2.7, ≤5.4, ≤3.9, <10, ≤5.3, and ≤3.6% for IL-15, follistatin, myostatin, FGF-21, irisin, resistin, and omentin, respectively, whereas the inter-assay variability was <9.1, ≤9.2, ≤6.0, ≤10.9, <15, ≤9.2, and ≤6.9%. The standard curves were analyzed by double parallel tube. The peak concentration and the area under the curve (AUC) displayed by the concentration-time data (trapezoid rule) were analyzed for each molecule. All the data were analyzed as a percent of baseline values.

### Statistical Analysis

Data are presented as mean ± SD unless otherwise stated. The normal distribution (Shapiro–Wilk test) and homoscedasticity (Levene’s test) of the data were checked before any statistical treatment. A two-way (condition [AT, FATmax], time) repeated-measures ANOVA was used to compare the response over time of the blood variables and RMR between the two types of exercise sessions (AT or FATmax). A Greenhouse–Geisser correction was applied when Mauchly’s test of sphericity was violated. Bonferroni *post hoc* test was conducted to analyze differences between conditions at each time point. To minimize the risk of statistical error type I, the level of significance was corrected for multiple comparisons by dividing 0.05 by the total number of time points (i.e., threshold *p*-value = 0.007 [0.05/7]). Student’s paired *t* tests were conducted to analyze the differences in energy expenditure and peak and AUC concentration between sessions. The magnitude of the differences between conditions was determined through the imputation of effect sizes (ES, Hedges’g). All statistical analyses were conducted using a statistical software package (SPSS 23.0, United States).

## Results

Subjects’ VO_2_max and maximum HR (HRmax) averaged 46 ± 4 ml kg min^−1^ and 192 ± 7 bpm, respectively. Although, all participants maintained the prescribed FATmax-associated velocity during the corresponding 45-min exercise bout an increase in RER could be observed in some of them by the end of exercise, with the subsequent variation in fat oxidation rate. By contrast, they could not exercise continuously for 45 min at the AT-associated velocity; accordingly, they were allowed to take one or two brief recovery periods (consisting of walking for 5 min at 3 km ⋅ h^−1^) before continuing running at the prescribed velocity until completing 45 min.

### Metabolic Response to Exercise

**FIGURE 1:**
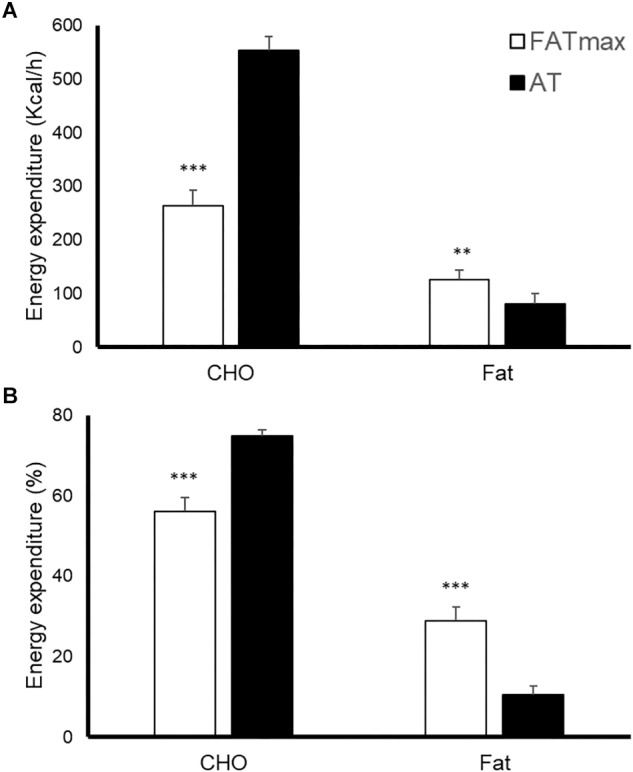
Energy expenditure [expressed as absolute **(A)** and relative **(B)** values] in response to 45-min exercise at the intensity of maximal fat oxidation (FATmax) or the anaerobic threshold (AT). Data are mean ± standard error. Significant differences between conditions: ^∗∗^*p* < 0.01, ^∗∗∗^*p* < 0.001. CHO, carbohydrate.

**FIGURE 2:**
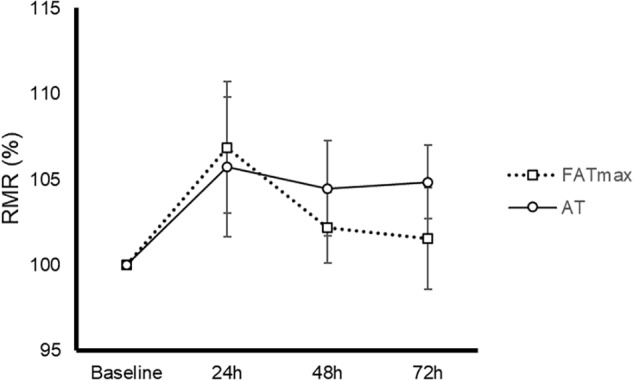
Time-course of resting metabolic rate (RMR) in response to 45-min exercise at the intensity of maximal fat oxidation (FATmax) or the AT. Data are mean ± standard error. No significant condition, time, or condition by time effect (*p* > 0.05) was found.

AT was performed at a higher intensity than FATmax (85 ± 8 vs. 52 ± 14% of VO_2 max_ and 90 ± 4 vs. 68 ± 9% of HRmax, both *p* < 0.001). As a consequence, AT resulted in a higher energy expenditure (762 ± 118 vs. 480 ± 160 kcal/h, *p* < 0.001). AT also induced a greater absolute and relative oxidation of carbohydrates, but a lower contribution of fat (Figure 1). No condition (*p* = 0.674), time (0.073) or condition by time effect (*p* = 0.664) was observed for post-exercise RMR (Figure 2).

### Blood Variables

**FIGURE 3:**
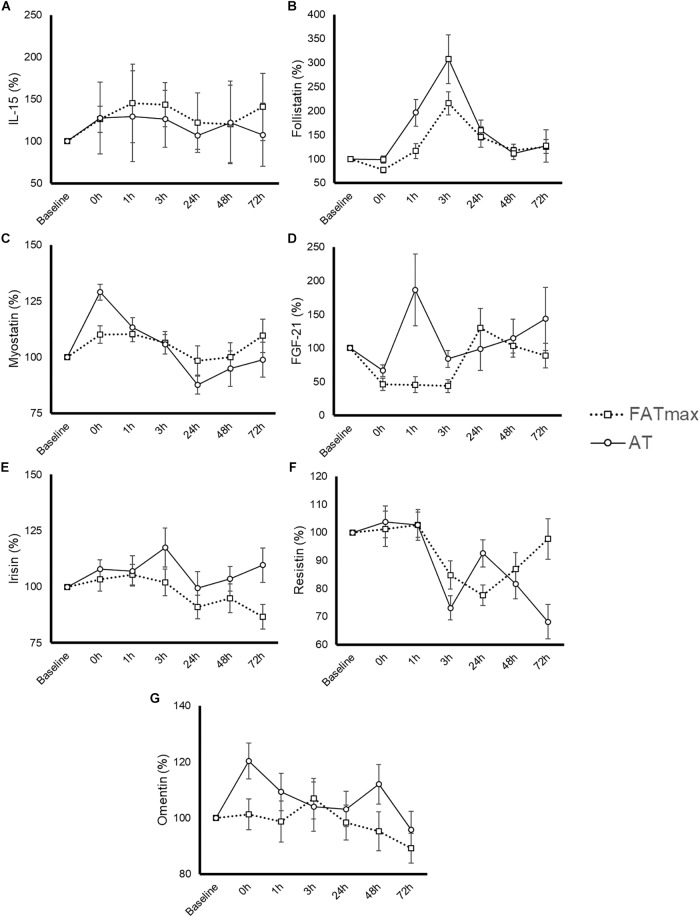
Time-course response of interleukin (IL)-15 **(A)**, follistatin **(B)**, myostatin **(C)**, fibroblast growth factor-21 (FGF-21) **(D)**, irisin **(E)**, resistin **(F)**, and omentin **(G)** to 45-min exercise at the intensity of maximal fat oxidation (FATmax) or the AT. Data are mean ± standard error. All molecules were measured in 14 subjects except for IL-15 and FGF-21, which were measured in 8 and 13 of them, respectively. The significance threshold for *post hoc* analyses was adjusted for the number of time points (i.e., *p* = 0.05/7 = 0.0071). A significant condition by time effect was observed for resistin and omentin, but there were no significant differences between conditions at any specific time point (*p* > 0.007).

The time course and peak and AUC concentrations for each biomarker are displayed in Figure 3 and Table 1, respectively.

**Table 1:** Myokine/adipokine response to aerobic exercise at the intensity of maximal fat oxidation (FATmax) or the anaerobic threshold (AT).

	Peak (%)				AUC (%)			
	FATmax	AT	*p*-value	ES	FATmax	AT	*p*-value	ES
IL-15	194 ± 118	160 ± 41	0.180	0.36	9251 ± 7374	8342 ± 6947	0.434	0.16
Follistatin	241 ± 86	359 ± 177	**0.024**	0.82	10302 ± 3451	11688 ± 3280	0.376	0.40
Myostatin	124 ± 23	136 ± 18	0.160	0.56	7382 ± 1540	6891 ± 1244	0.374	0.34
FGF-21	155 ± 92	294 ± 209	**0.009**	0.83	7073 ± 3464	7982 ± 4877	0.589	0.21
Irisin	116 ± 19	131 ± 30	0.121	0.58	6746 ± 1292	7603 ± 1661	0.081	0.56
Resistin	66 ± 13	57 ± 13	0.073	0.56	6184 ± 923	5902 ± 801	0.375	0.21
Omentin	114 ± 24	129 ± 24	0.071	0.75	6997 ± 1621	7582 ± 1735	0.283	0.44

*Data are mean ± SD and % of baseline values. All molecules were measured in 14 subjects except for IL-15 and FGF-21, which were measured in 8 and 13 of them, respectively. Contrary to the other myokines/adipokines, peak concentration for resistin represents the lowest value recorded. Significant p-values (p < 0.05) are highlighted in bold. AT, anaerobic threshold; AUC, area under the curve concentration; ES, effect size; FGF-21, fibroblast growth factor-21; and IL-15, interleukin-15.*

#### Interleukin-15

Six participants presented a IL-15 concentration below the minimum detection levels and thus their results could not be analyzed (total *n* for analyses = 8 per condition). No significant condition (*p* = 0.435), time (*p* = 0.550) or condition by time effect (*p* = 0.905) was observed for IL-15 (Figure 3A), with no differences in peak or AUC concentration between conditions (Table 1). No significant changes in IL-15 levels were observed at any time point after AT or FATmax exercise compared with baseline.

#### Follistatin

A significant time (*p* < 0.0001 but no condition (*p* = 0.073) or condition by time effect (*p* = 0.164) was observed for follistatin (Figure 3B). A trend to an increase in follistatin levels above baseline values was observed up to 3 h after both AT and FATmax exercise (*p* = 0.026 and 0.007, respectively but above the corrected threshold *p*-value of 0.007). However, a higher peak follistatin was observed after the AT session, with no differences between conditions in AUC (Table 1).

#### Myostatin

A significant time (*p* < 0.0001) but no condition (*p* = 0.870) or condition by time effect (*p* = 0.067) was observed for myostatin (Figure 3C), with no differences between conditions in peak or AUC concentration (Table 1). No changes were observed in myostatin levels after FATmax exercise compared to baseline, but a significant increase was observed immediately after the AT session (*p* < 0.001).

#### Fibroblast Growth Factor-21

One participant presented with a FGF-21 concentration below the minimum detection levels and thus his results could not be analyzed (total *n* for analyses = 13). A significant condition effect (*p* = 0.009) and a trend to a significant time effect (*p* = 0.061) was observed for FGF-21 (Figure 3D). A trend toward an increase in FGF-21 levels above baseline values was observed immediately after AT exercise (*p* = 0.059). By contrast, FGF-21 levels decreased above baseline values at 0 (*p* = 0.002), 1 (*p* = 0.013, yet above the threshold *p*-value) and 3 h (*p* = 0.001) after the FATmax session, respectively. We found no condition by time effect (*p* = 0.112), but a greater peak concentration was observed after AT exercise (Table 1). There were no differences between conditions in AUC concentration (Table 1).

#### Irisin

A significant time effect (*p* = 0.011) and a trend toward a significant condition by time effect (*p* = 0.067) was observed for irisin (Figure 3E). However, there were no differences between conditions in peak or AUC concentration (Table 1). No significant changes in irisin levels were observed at any time point after AT or FATmax exercise AT compared to baseline.

#### Resistin

A significant time (*p* < 0.001) and condition by time effect (*p* < 0.001) was observed for resistin. Resistin levels were significantly decreased 24 h after FATmax exercise compared to baseline (*p* = 0.001). In turn, they were significantly decreased 3 (*p* = 0.001) and 72 h (*p* = 0.004) after AT compared to baseline, and a trend toward lower levels was also found 48 h post-exercise (*p* = 0.086). No significant differences were found between conditions at any specific time point (Figure 3F). No differences between conditions were found in AUC concentration, but a trend toward a lower minimal concentration was found after AT compared to FATmax (Table 1).

#### Omentin

A significant time (*p* = 0.033) and condition by time effect (*p* = 0.031) was observed for omentin. *Post hoc* analysis revealed a trend toward higher values with AT compared to FATmax immediately after exercise (post 0 h, *p* = 0.020) as well as toward higher values 48 h later (*p* = 0.070) (Figure 3G). There were no differences between conditions in AUC concentration, but a trend toward a higher peak concentration was observed after AT compared to FATmax (*p* = 0.071) (Table 1).

## Discussion

The present study compared the response of several myokines/adipokines involved in metabolic regulation and weight management after two exercise sessions performed at the AT or FATmax AT (at mean intensities corresponding to ∼85 and ∼52% of VO_2_max, respectively) in healthy subjects. We expected *a priori* to observe a greater response with the latter given that it was performed at a considerably higher intensity. In this respect, our results indeed showed that AT induced a ∼2 and ∼1.5-fold higher increase in FGF-21 and follistatin concentration, respectively, compared to FATmax, as well as a greater (20%) omentin response immediately post-exercise. However, no significant differences were observed for the rest of myokines/adipokines. No differences were found either in the time course of RMR post-exercise.

Previous research has analyzed the myokine/adipokine response to exercise. However, the influence of manipulating training variables such as exercise load on this response remains to be elucidated, and there is scarce information on the time-course of this response. In this respect, we recently observed a high individual variability in the myokine response to two popular exercise modalities such as high-intensity interval training (commonly known as “HIIT”) and resistance training (He et al., 2018). In the present study, however, we assessed the myokine/adipokine response of the same individuals to two sessions of the same type of exercise (i.e., “aerobic” or “endurance”) but performed at different relative intensities and thereby resulting in different total exercise loads. This design allowed us to study the role of exercise load on this response. To our knowledge, this is the first study to assess how manipulating training load modifies the myokine/adipokine response to exercise. Moreover, here we described the time-course response of these factors up to 3 days after exercise, which might help to gain insight into the duration of the metabolic effects of a single exercise session. The observed trend to an exercise-induced increase in FGF-21 and follistatin levels is in agreement with previous research (Hansen et al., 2011; Kim K. H. et al., 2013; Tanimura et al., 2016). FGF-21 regulates glucose homeostasis and lipid utilization, augments brown fat thermogenesis, and has been related to improved insulin resistance, weight loss and to the browning of WAT (Lee et al., 2012, 2014; Woo et al., 2013; Fisher and Maratos-Flier, 2016). In turn, follistatin is a myostatin-binding protein that promotes skeletal muscle development through the activation of the mammalian target of rapamycin pathway (Winbanks et al., 2012). Follistatin also plays a role in metabolism (i.e., reduction of body fat, improvement of glucose homeostasis and WAT browning) (Braga et al., 2014). Thus, increases in these two myokines could be potentially related to some of the cardiometabolic health benefits usually obtained with exercise, and our results suggest that AT might be more effective for this purpose than FATmax.

Increasing exercise intensity also resulted in higher circulating levels of omentin, an adipokine that seems to counteract insulin resistance and obesity (De Souza Batista et al., 2007), improving glucose metabolism through the stimulation of Akt phosphorylation in muscle tissue (Yang, 2006). In line with these results, 12 weeks of aerobic exercise have proven superior to other types of exercise (i.e., resistance exercise and a combination of both resistance and aerobic exercise) for the promotion of omentin production in diabetic animals (de Castro et al., 2019). Similarly, 12 weeks of aerobic exercise have also been reported to result in improved cardiometabolic risk factors (waist circumference, insulin resistance, and lipid profile) together with increased circulating omentin levels in overweight/obese men (Saremi et al., 2010), which has been corroborated in later studies (Wilms et al., 2015; Ouerghi et al., 2017). Endurance exercise appears therefore as an effective strategy for the stimulation of omentin release by adipose tissue – with subsequent potential metabolic benefits – but the present findings suggest that the magnitude of these benefits might depend on exercise intensity.

Although no differences were observed between conditions for the myostatin response, only AT induced a transient increase of this protein immediately after exercise. Myostatin, a transforming growth factor (TGF)β family member and the first described myokine, is a negative regulator of muscle growth (Huang et al., 2011). Several studies have reported a down-regulation of myostatin expression after both endurance and resistance exercise (Louis et al., 2007; Hittel et al., 2010; Lundberg et al., 2012). In contrast, other authors found an increase in myostatin mRNA in contracting muscles after exercise, which was coupled to a decrease in its transcriptional activity due to the activation of Notch, a TGFβ inhibitor (MacKenzie et al., 2013). Thus, although one could hypothesize that the increase in myostatin observed after AT might reduce the anabolic response to exercise, increases in myostatin expression *per se* should not necessarily result in lower skeletal muscle hypertrophy.

By contrast, exercise induced no significant increases in one of the most studied myokines, IL-15 (Fiuza-Luces et al., 2018). IL-15 has been purported to have beneficial effects on metabolic homeostasis through a decrease in WAT (Nielsen et al., 2008) and an enhancement of glucose metabolism (Busquets et al., 2006; Kim H. J. et al., 2013). Previous studies have found acute increases in the circulating levels of this myokine after resistance exercise training (Riechman, 2004; Pérez-López et al., 2018), but mixed results have been reported regarding the effects of endurance exercise. In agreement with our findings, no changes in plasma levels of IL-15 were observed after 1 (Chan, 2004) or 3 h of cycling exercise (Rinnov et al., 2014), or after 2.5 h of running (Ostrowski et al., 1998). On the other hand, Tamura et al observed an immediate increase in IL-15 plasma levels in response to 30 min of treadmill running, although IL-15 returned to basal values 3 h later (Tamura et al., 2011). There is therefore controversy on whether endurance exercise stimulates the release of IL-15 from muscles to the bloodstream, and more research is warranted to elucidate potential factors that might influence the production of this myokine.

Both training sessions decreased resistin levels at least at one time point post-exercise, and although the minimum values registered tended to be lower after AT compared to FATmax, no consistent differences between sessions were found. Resistin is produced by WAT and brown adipose tissue, antagonizes insulin action, and is elevated in obesity (Steppan et al., 2001; Adeghate, 2004). In agreement with our findings, plasma resistin levels have been reported to decrease acutely after resistance training in sedentary post-menopausal women (24 and 48 h post-exercise), and reduced values of this adipokine were also found when this training program was maintained for 16 weeks (Prestes et al., 2009). Other authors have found decreased resistin levels after long-term aerobic exercise training (Kadoglou et al., 2007; Jones et al., 2009). By contrast, Jamurtas et al. (2006) showed no changes in plasma resistin levels up to 48 h after a 45-min bout of cycling at 65% of VO_2_max, and no variations in the levels of this adipokine were found after 14 weeks of exercise with or without concomitant diet in post-menopausal women with type 2 diabetes (Giannopoulou et al., 2005). More research is thus needed to determine if intensity mediates exercise effects on this adipokine.

No exercise effects or differences between conditions were observed for irisin. Despite the attention given to irisin in recent years owing to its advocated role in WAT browning and energy expenditure (Pedersen and Febbraio, 2012; Elbelt et al., 2013), controversy exists on the biological relevance of this myokine (Raschke et al., 2013), and some methodological issues regarding its measurement have been raised (Albrecht et al., 2015). Further research is needed to confirm the effect of exercise on irisin concentration and the potential of this myokine as a therapeutic target against obesity and its related complications.

The present study has some limitations that must be noted, notably the lack of measurement of some important myokines such as IL-6. Moreover, although our findings suggest that AT might induce a greater peak response to exercise than FATmax in some myokines, we cannot discern if these differences would eventually translate into actual benefits in cardiometabolic health. Finally, the present results, obtained in young healthy individuals, might not be necessarily applicable to other populations (e.g., obese or older people).

In summary, a higher endurance exercise intensity (i.e., AT vs. FATmax) induced a higher response of follistatin, FGF-21 and omentin in healthy subjects, which could have potential implications due to the role of these myokines/adipokines in muscle plasticity and metabolism. However, no consistent differences between sessions were observed for IL-15, myostatin, irisin, or resistin. To optimize exercise prescription future research should determine the effects of varying exercise loads in the myokine/adipokine response of other populations (e.g., including people with cardiometabolic conditions) and to assess whether manipulating other exercise variables such as exercise type or volume might increase the time-course response of myokines/adipokines.

## Ethics Statement

Participants signed an informed consent form after having the procedures explained. The study was conducted in accordance with the Declaration of Helsinki and was approved by the Institutional Review Board (Ethics Committee of the Chinese Institute of Sport Science).

## Author Contributions

ZihH, YT, CH, JZ, PH, ZilH and SY conceived the study and performed the experiments. PV and AL analyzed the data and drafted the manuscript. All authors significantly contributed to the final version of the manuscript.

## Conflict of Interest Statement

The authors declare that the research was conducted in the absence of any commercial or financial relationships that could be construed as a potential conflict of interest.
